# Modelling of dark fermentation of glucose and sour cabbage

**DOI:** 10.1016/j.heliyon.2021.e07690

**Published:** 2021-07-31

**Authors:** Gaweł Sołowski, Krzysztof Pastuszak

**Affiliations:** a Gdansk University of Technology, Faculty of Mechanical Engineering, Poland; b Gdansk University of Technology, Department of Algorithms and Systems Modelling, Faculty of Electronics, Telecommunications and Informatics, Building A, EA 226, Poland

**Keywords:** Hydrogen production, Modelling, Stress parameters, Bacteria, Low pH, Arcus tangent

## Abstract

In the article, modified Anaerobic Digestion Models 1 (ADM-1) was tested for modelling dark fermentation for hydrogen production. The model refitting was done with the Euler method. The new model was based on sets of differential equations. The model was checked for hydrogen production from sour cabbage in batch and semi-batch in 5 g VSS (volatile solid suspension)/L and at the semi-batch process from glucose at 5 and 10 g VSS/L. Added parameters determined the conversion of a substrate, hydrogen production, and stress parameters. In the case of a semi-batch process, for one month, cumulative hydrogen production from sour cabbage of 5 g VSS/L was 0.9 L of cumulative hydrogen volume and from glucose 5 g VSS/L (in case of feeding 2 g VSS/L every two days) 2.5 L of cumulative hydrogen volume. At the bacterial population level, hydrogen production was a continuous process at an adequate range of population size and environmental parameters.

## 1 Introduction

Hydrogen is still mainly produced by conventional methods from fossil fuels, despite being considered the biofuel of the future [1]. Therefore, in the transformation times from non-renewable methods to the more sustainable ones, researchers seek efficient hydrogen production approaches from water and biomass (renewable only if people number is below 10 bln) [2]. Anaerobic digestion (AD) that stops at acidogenesis with a shift to hydrogen production is called dark fermentation (DF). The transformation occurs if the inoculum is pretreated by heat or other stress factors [3]. During the first hours of anaerobic digestion, an excess of hydrogen is often observed [4]. Later this excess does not occur, consumed completely, after acidogenesis by methane production [5]. The process can be provided by psychrophilic from 15 °C to 30 °C [6], mesophilic from 33 °C to 40 °C) [7, 8], and thermophilic conditions from 55 °C to 80 °C [9]. The optimal pH conditions for dark fermentation are in a range from 5.0 to 6.0 [10]. The most challenging problem is the process design and choice of the selection method to determine proper substrates, an appropriate strain of bacteria, and convenient thermal and chemical conditions [11]. In the case of substrate potential evaluation, some assessment methods already exist [12], e.g., dark fermentation equivalent of Buswell equation [13]. Gompertz equation was introduced for empirical results calculation for the bacterial growth under dark fermentation conditions [14]. Several attempts were made to estimate the hydrogen production by DF, like ANN [15] approaches to modelling bacterial hydrogen production [16], and as presented by Pan et al. [17]. All these methods were tested and worked only for glucose [18]. Glucose is a too expensive material for use as a substrate for dark fermentation at the industrial scale. Another model used trigonometric function for palm oil effluent DF (only tested more complex substrate [19]). Anaerobic Digestion Models 1 (ADM-1) is the most commonly used model in anaerobic digestion [20]. ADM-1 models are relationships between bacterial growth, a decline in substrate concentration, and accumulated methane production increase in biogas [21]. The ADM-1 kinetics is based on the Monod equations and Luedeking Piret Model [22]. AD is a derivative of dark fermentation. The model based on ADM-1, proposed by Markowski et al. [21] was tested for glucose and sour cabbage in mesophilic conditions most commonly used [23]. The updated model involves different parameters: pH [24], concentration, stress [25], and micro-aeration [26]. The study aims to modify the ADM-1 model to form DFM-1 (Dark fermentation Model 1), thus providing a method for modelling the growth of bacteria, with the computation of cumulative hydrogen production using numerical methods. The model used successful data from sour cabbage for 5 g VSS/L [27]. Then the model verified new semi-batch experiments of sour cabbage and glucose.

## 2 Materials and methods

### 2.1 Mathematical model

Mathematical modelling and optimization were performed using Matlab R2017® on a supercomputer Tryton from Academic Computing Centre TASK in the Gdansk University of Technology. The computations required 11 central processing units and 12 GB of RAM. The modeling was based on a set of differential equations and solved using the Euler method with a time step of 0.1 h, which proved experimentally to be sufficiently small to minimize the truncation error. The model was based on ADM-1 [28] and the palm oil model [19] and fit experimental data at two glucose and sour cabbage concentration levels (5 g VSS/L and 10 g VSS/L). Based on literature data [29] space of feasible solutions was determined. An exhaustive grid search minimizing objective function was applied to determine the parameters. Local grid search around computed values adjusted further the parameters. Mean squared error (MSE) was used as the objective function. All model is explained as part of results in section Theory and Calculation.

### 2.2 Empirical validation

**Figure 1:**
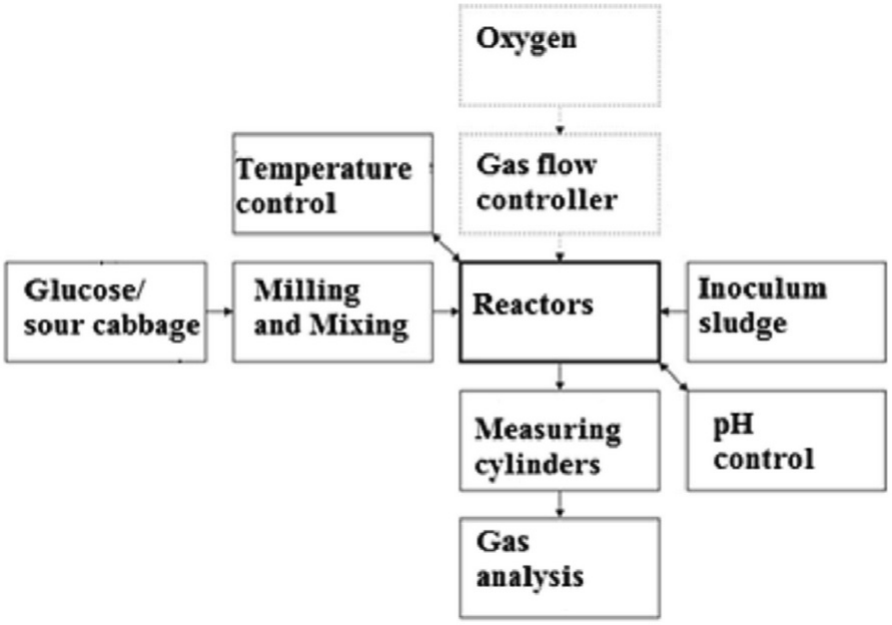
Sketch of the experimental procedure for hydrogen production by dark fermentation of sour cabbage/glucose.

**Figure 2:**
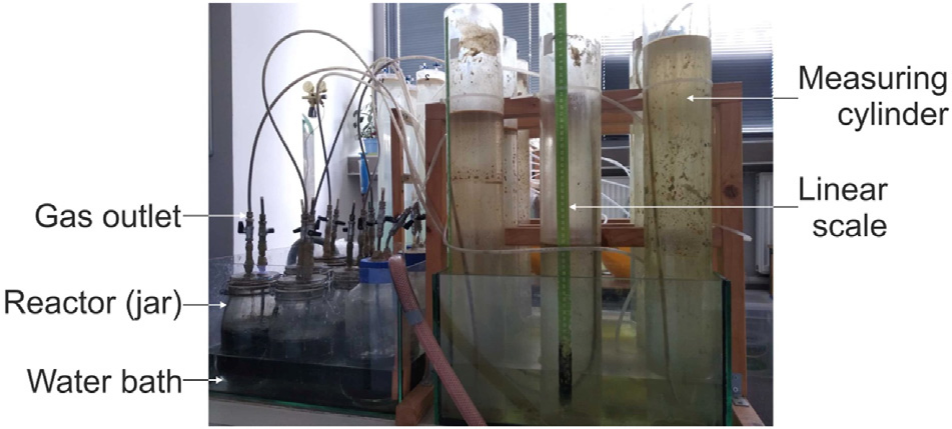
Photo of the experimental setup.

The fermentation process of sour cabbage was performed in reactors of volumes 2 dm^3^ with working volume 1.2 dm^3^ (Figures 1 and 2). Figure 1 shows a sketch of the experimental procedure for hydrogen production by DF of sour cabbage and glucose. The bacteria layer was a sludge from a biogas plant in Darżyno near Gdansk. The inoculum used for experiments came from a mesophilic digester treating mainly maize silage and pig manure. The boiled inoculum was prepared in the investigation to show changes in bacteria growth due to the description of Nasirian et al. [30]. Digesters were kept in a water bath under mesophilic conditions (38 ± 2 °C) during the process (for maintaining proper temperature). Before fermentation, the batch reactors (see Figure 2) were flushed with nitrogen to maintain strictly anaerobic conditions at the beginning of the process. The gas produced by every fermenter was collected in a cylindrical vessel filled with water and a barrier liquid. The water for eliminating carbon dioxide dissolving was on top marked with the mixture by mass 1:10 detergents for dishes (Ludwik®) and diesel oil. The sour cabbage before the introduction to digestors was milled and mixed. Semi-batch reactors were fed with anhydrous glucose powder (for glucose tolerance test). In the semi-batch, 5 g VSS/L of sour cabbage or glucose was added to the inoculum. Every set of experiments was triplicate, the results were mean given are mean values similar to [31]. After addition, the pH value for the acidic value of the mixture was lowered (using 38% HCl) from 7.9 to 5.0. In the case of unboiled inoculum and sour cabbage was adjusted by the previous procedure to pH 6.0. In sour cabbage there is also implemented micro-aeration of oxygen flow rate (OFR): 0.58 mL/h (pH 6.0 and raw inoculum), 0.63 mL/h (pH 7.5 and raw inoculum), 0.8 mL/h (pH 5.0 boiled, semi-batch). Sour cabbage was milled to a size suitable for feeding with a syringe used for micro-aeration. After closing, the reactors were purged with nitrogen for 5 min to remove oxygen and then microaerated. Reactors were microaerated twice per day using a syringe of 25 mL volume with error ±0.1 mL until the fermentation process stopped. The feeding for sour cabbage was every six days, with a portion of 3 g VSS/L. Glucose feeding was: 3 g VSS/L every three days for the initial concentration of 10 VSS/L. In glucose concentration of 5 g VSS/L, feedings were: 2 g VSS/L every two days, and 2g VSS/L every three days. Table 1 displays the characteristics of substrates and inoculum. Substrate and bacteria characteristics were determined using, dry mass (total solid TS) dry organic mass (volatile suspended solid) parameters according to [32]. Substrates evaluated for dark fermentation purposes were sour cabbage and glucose. The glucose and cabbage concentration used were 5 g VSS/L. Additionally, for glucose 10 g VSS/L was also tested.

**Table 1:** Characteristics of biomass.

Material	pH	TS	VSS
Inoculum raw (without pretreatment)	8.24	1.09% ± 0.028%	37.44% TS± 1.03%
Inoculum boiled (After heat shock)	7.84	1.5% ± 0.03%	37.91% TS ± 1.22%
Glucose	5.3	98% ± 0.03%	78% TS ± 0.77%
Sour Cabbage	4.61	6.99% ± 0.02%	89.32%TS ± 1.2%

Empirical data was used to determine parameters such as growth index, Monod constants, and inhibition constant. Qualitative and quantitative determinations were performed using gas chromatography (GC-TCD) with a thermal conductivity detector (TCD) and argon as a carrier gas. The Silco packed column Restek® of characteristics 2m/2mm ID 1/8″ OD Silica was used. Hydrogen, methane, carbon dioxide, and nitrogen, were determined at a flow rate of 0.6 mL/h.

The volatile acid analysis is provided by Ekotechlab lab with characteristics in Table 2.

**Table 2:** Characteristics of volatile acid contamination and determination of compounds.

Technique and method:	Volatile acids contamination in sample using (GC-FID)
Equipment:	Gas chromatograph Thermo Scientific Trace 1300
Analysis conditions:	Column: Rxi 5MS 60m Gas carrier: helium Flow: 1.0 ml/min The temperature of injection: 250 °C Stream separation: 1:10 Detector FID: 300 °C Temperature program: from 40 °C (3 min) - 20 °C/min to 300 °C–300 °C (5 min)
Sample preparation:	To sample (6 mL) sulphuric acid (VI) (drop 0.25 mL) and sodium chloride (100 mg), then extracted with tert-butyl-methyl ether (2 mL)
Technique and method:	Determination of compound in gas chromatograph with a mass spectrometer (GC-MS)
Equipment:	Gas chromatograph of firm Shimadzu GC-2010Plus
Analysis conditions:	Column: Rxi 5MS 60m Gas carrier: helium Flow: 1.0 mL/min The temperature of injection: 250 °C Stream separation: 1:20 Detector MS: 210 °C Temperature program: from 50 °C (4 min) - 20 °C/min to 300 °C–300 °C (5 min)
Sample preparation:	To sample (6 mL) sulphuric acid (VI) (drop 0.25 mL) and sodium chloride (100 mg), then extracted with tert-butyl-methyl ether (2 mL)

## 3 Theory and calculation

The model was formulated using the ADM-1 for methane production transformed into a hydrogen production model. The initial point for model construction was a set of equations proposed by Markowski et al. [33]. The Markowski Model consists of a set of 3 differential equations – (1–3).

(1) $$\frac{dX}{dt}={\text{μ}}_{\text{max}}\frac{\text{S}}{{\text{K}}_{\text{Max}}+\text{S}}\text{X}\frac{{\text{S}}^{\text{a}}}{{\text{K}}_{\text{I}}+{\text{S}}^{\text{a}}}$$

(2) $$\frac{dS}{dT}=w\frac{dX}{dT};$$

(3) $$\frac{dP}{dT}=z\frac{dX}{dt}\text{s}$$

where:X – bacteria cell concentration g VSS/L;
S – substrate concentration g VSS/L;
P – cumulative methane volume L;
a - inhibition parameter from 1 to 2;
K_max_ – Monod constant for growth;
K_I_ - Monod constant of inhibition;
w - yield coefficient of differences of a substrate to cell concentration constant in case of anaerobic digestion;
z - yield coefficient of differences in the volume of methane to cell concentration;
μ_Max_ - maximal bacterial growth.

Methane production is a derivative of acetogenesis, in which the hydrogen production process occurs. Computations required proper function selections for model kinetics and numerical schemes to solve the model for kinetic parameters. The high dimensionality of solution space required a number of a priori assumptions. K_max_ and K_I_ values were calculated using the Luedeking Piret equation. Both constants K_max_ and K_I_ were calculated as 5 g VSS/L. The maximal bacteria growth from Monod was determined as 4.89 g/L. For the formulation of the final model based on ADM-1, the answering to the following questions was necessary:

• whether the inhibition of hydrogen production was dependant on their prior stress occurrence;
• whether for sour cabbage pH range should be considered - low pH 5.0 was assumed to be the most suitable.

Determination of the expected shape of hydrogen production curves was based on experimental results. The evaluation suggested that cumulative hydrogen production should resemble the shape of the arctan function as shown in Figure 2. Hence the function under the derivative of cumulative production should give dx/(1 + x).

DF was only the butyric pathway [34] due to Ekotechlab analysis. In the first cycle of the pathway one hydrogen molecule and pyruvate are produced, and then a second hydrogen molecule [10]. The model aimed to check group behavior to find continuity of the process. Hydrogen production from bacteria was a metabolism result [35]- one unit periodically consumed substrate, reproduced (cell division), and produced hydrogen – hence for one bacteria, this was not a continuous process that could be described by differential equations. Therefore, analyses were performed at the level of groups of bacterial cells rather than single cells. The process could appear continuous at enough numerous range of population size and environmental parameters. Therefore, the substrate consumption was divided into two phases: S as substrate concentration and H as pyruvate concentration. See reactions 1 and 2 [10]; Eq. (4), pyruvate conversion from the substrate was assumed as 80%.(4)
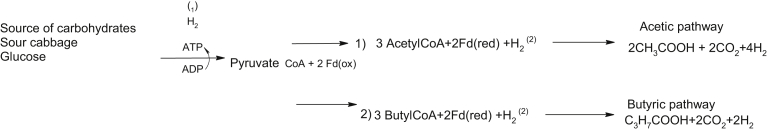


The presented modification of Markowski's model accounts for the division of processes into phases, both of which occur concurrently in the population (although not concurrent in the same bacteria cell). Hence hydrogen in a mathematical sense was an integration of the cumulative methane process P was here hydrogen. Thus for the investigated case, the Markowski model was as follows:

(5) $$\frac{dX}{dt}={\text{μ}}_{max}\frac{S}{K_{Max}+S}X\frac{S^{a}}{K_{I}+S^{a}}$$

(6) $$\frac{dU}{dt}={\text{μ}}_{max}\frac{H}{K_{Max}+H}X\frac{H^{a}}{K_{I}+S^{a}}$$

(7) $$\frac{dS}{dT}=w\frac{dX}{dT};$$

(8) $$\frac{dH}{dT}=ww\frac{dU}{dT};$$

(9) $$\frac{dP}{dT}=z\cdot \frac{dX}{dt}S-zz\frac{dU}{dt}H$$

The Euler method applied to the model resulted in a set of 5 equations. Resulted from the change as follows:

(10) $${\frac{dX}{dt}=((\left(\alpha \text{S}\cdot S\left(\text{t}\right)\right)/\left(m+S\left(t\right)\right)\frac{S{\left(t\right)}^{d}}{k+S{\left(t\right)}^{d}}X(t))}^{\kappa A}+\left(\left(\alpha \text{S}\cdot S\left(\text{t}\right)\right)/\left(m+S\left(t\right)\right)\;〖S(t){〗}^{d}/\left(k+〖S(t){〗}^{d}\right)〖X(t{〗}^{\xi A}\right)acot\left(ϙ\pi \right)q$$

(11) $$\frac{dU}{dt}=\left(\left(\lambda U\left(t\right)\frac{H\left(t\right)}{m+H\left(t\right)}\cdot \frac{H{\left(t\right)}^{d}}{k+H{\left(t\right)}^{d}}\right)|\omega +{\left(U\left(t\right)\frac{H\left(t\right)}{m+H\left(t\right)}\cdot \frac{H{\left(t\right)}^{d}}{k+H{\left(t\right)}^{d}}\right)}^{\tau }\right)acot\left(ϙ\pi \right)q$$

(12) $$\frac{dS}{dT}=w\frac{dX}{dT}$$

(13) $$\frac{dH}{dT}=ww\frac{dU}{dt}$$

(14) $$\frac{dP}{dT}=\left(\frac{sRate}{S}z\frac{dX}{dt}\right)-\left(\frac{hRate}{H}zz\frac{dU}{dt}\right)$$

where:

• X – first group of bacteria that takes substrate and produces hydrogen g VSS/L;
• S – substrate initial concentration g VSS/L;
• P – cumulative hydrogen volume L;
• αS – coefficient of conversion of a substrate;
• d, ξA, κA, ω, τ ϙ – stress coefficients; changes due to adding oxygen, heat shock pH change;
• U –the second group of bacteria that takes digested substrate from the first stage and produce hydrogen but less that it takes for a process;
• Q - maximal growth of bacteria calculated from Monod as 4.89 g/L;
• m, k – Monod constants 5 g/L;
• H - converted substrate S for U bacteria assumed as 0.78 of S, g/L;
• λ – bacteria coefficient for U bacteria;
• W - coefficient of conversion of substrate S by the bacteria X – analyzed in range: (-2:0; 0.5);
• ww – coefficient of conversion of substrate H by bacteria U – analyzed in the range: (-1:0; 0.5);
• z - coefficient of the relation between the produced hydrogen and bacteria X – range: (0:3; 0.06);
• zz - coefficient of the relation between the produced hydrogen and bacteria U (0:3; 0.06);
• hRate, sRate - other stress coefficients related to H and S: hRate in range (0.6:2.6; 0.01); sRate in range (0.3:0.9; 0.01).

When optimization schemes such as the Levenberg-Marquardt algorithm proved to give an inaccurate fit, an exhaustive grid search was performed. Computed values that globally minimized MSE in the considered parameter space were then further adjusted using local grid searches at higher resolutions. The non-pretreated inoculum (raw) and pretreated (boiled) inoculum displayed significantly different behaviour. The numerical quantities of parameters are presented in the supplemental material at the end of the article (supplemental materials 21b.pdf). The parameters ww was constant while w was variable in the time.

## 4 Results and discussion

### 4.1 Empirical results

**Figure 3:**
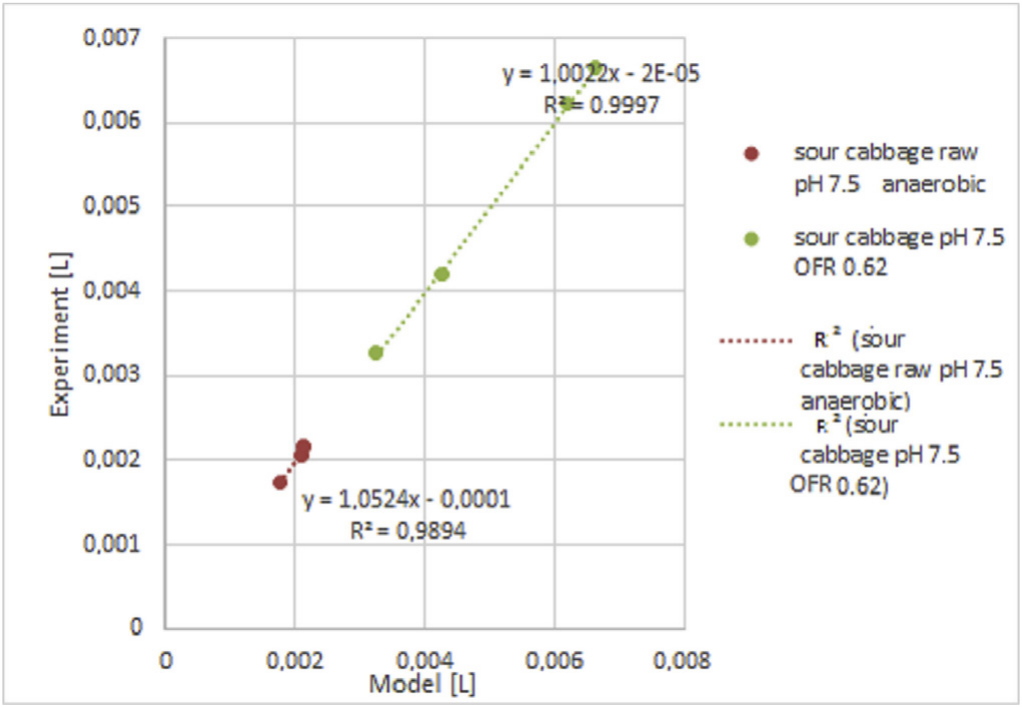
Cumulative hydrogen production model results vs Cumulative hydrogen production experimental results 5 g VSS/L at raw inoculum pH 7.5 different OFR [12].

**Figure 4:**
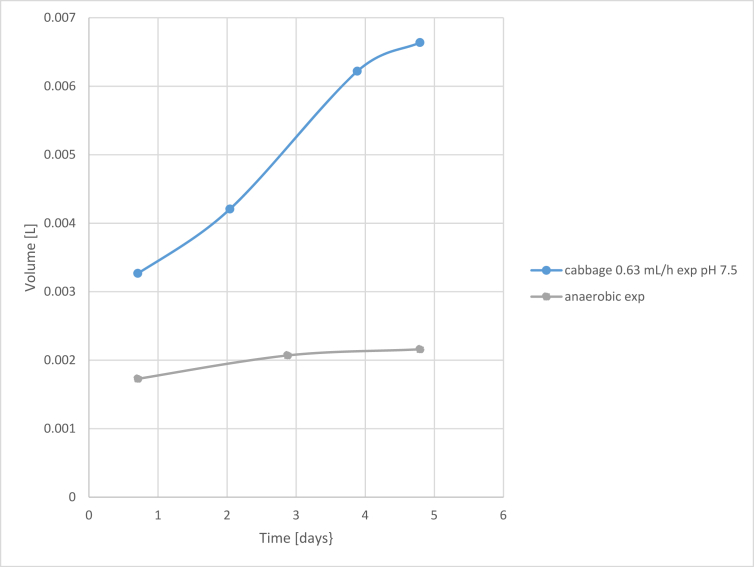
Cumulative hydrogen production model vs experiment 5 g VSS/L at raw inoculum pH 7.5 different OFR [12].

**Figure 5:**
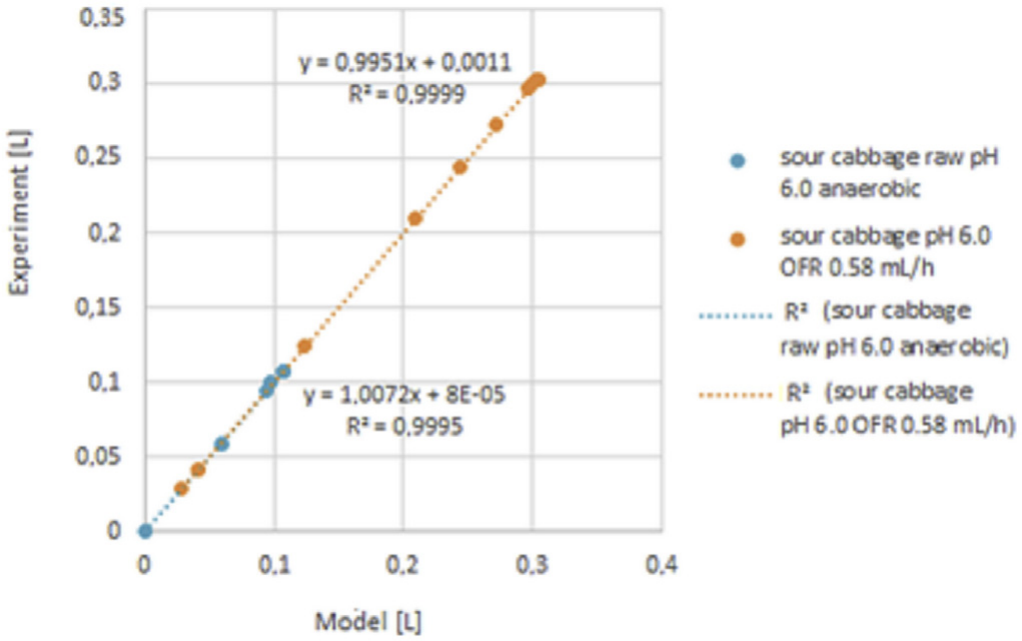
Cumulative hydrogen production experimental results vs model results from sour cabbage from raw inoculum at pH 6.0 for concentration 5 g VSS/L at different OFR.

In sour cabbage, hydrogen was produced in the case of raw inoculum for five days in neutral pH (Figure 3) [36] and 25 days in the case of pH 6.0 (Figure 4). Experimental and model results were compared for strictly anaerobic and optimum OFR. Due to significant differences, results were presented in two Figures. Then, the model was tested for semi-batch processes of glucose and sour cabbage. For glucose and sour cabbage of 5 g VSS/L were determined in detailed parameters (see Figure 5 and supplementary materials). The model precision was acceptable when differences between model results and experiments were smaller than 0.02 mL of hydrogen. The discussion part of this study described empirical and theoretical results analysis.

**Figure 6:**
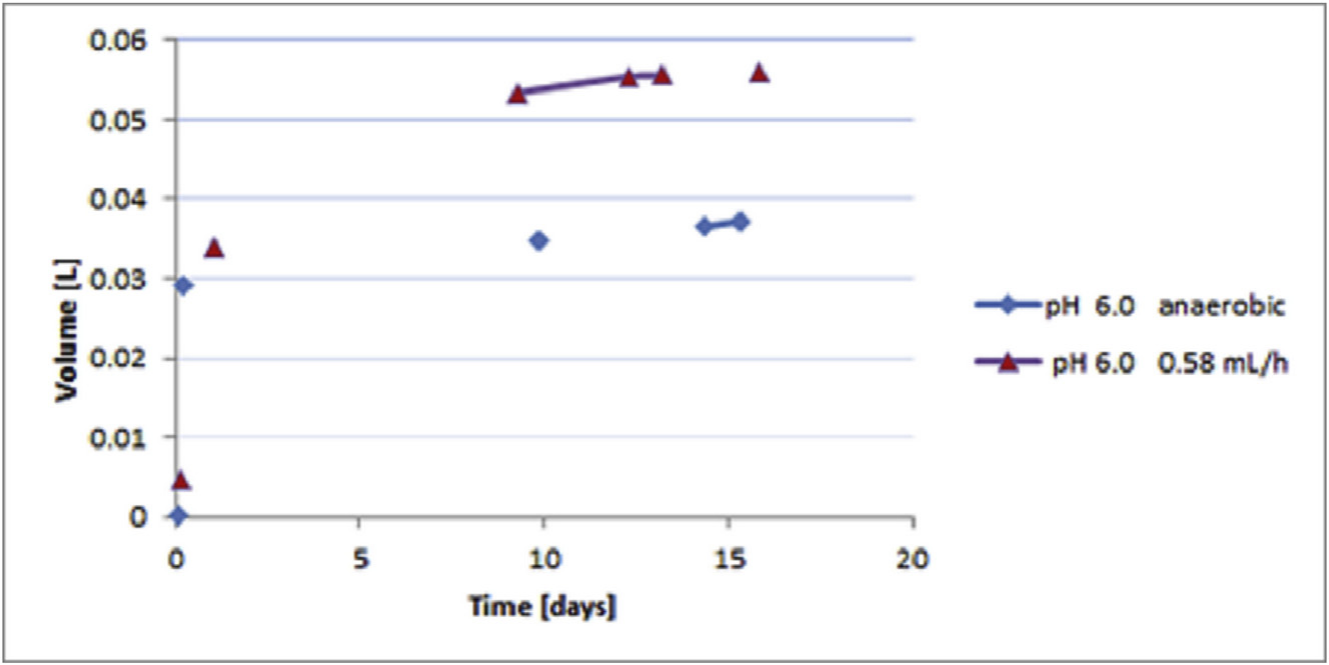
Cumulative hydrogen production experimental results from sour cabbage from raw inoculum at pH 6.0 for concentration 5 g VSS/L at different OFR.

**Figure 7:**
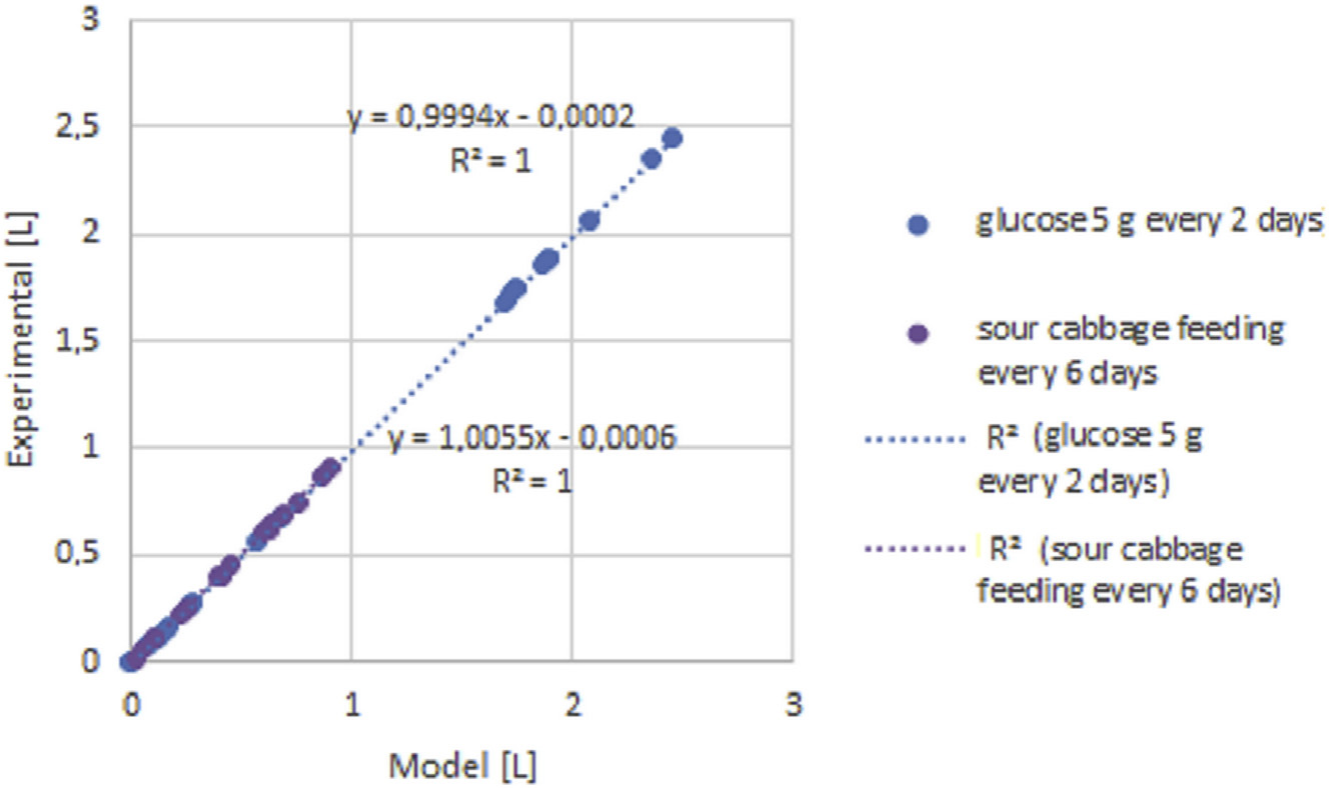
Cumulative hydrogen production model vs experiment results in semi-batch from glucose and sour cabbage models and experiments.

Figures show results of experiment and modelling. The semi-batch process provides more days of measurable hydrogen volume. Thus more points and more error is generating through modelling was done. The trouble with not boiled inoculum is that it produces short-lasting hydrogen production of measurable volumes in few times. Therefore few points are more precisely determined than in the case of continuous hydrogen production presented in Figures 6 and 7. Processes of short hydrogen production are relevant for showing limiting conditions in which hydrogen generation starts.

### 4.2 Discussion

**Figure 8:**
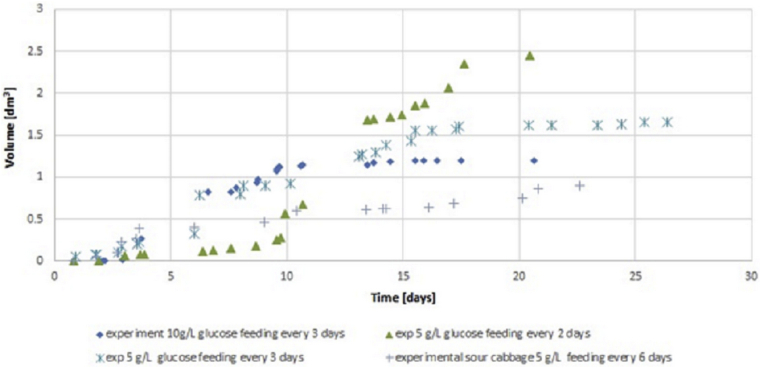
Cumulative hydrogen production experimental results in semi-batch from glucose (5 g VSS/L and 10 g VSS/L) and sour cabbage (5 g VSS/L).

Lowering pH to 5.0 improved hydrogen production in agreement with DF results (Figures 3 and 4) [37]. The points meaning that biogas production was above 0.4 L. In the case of anaerobic, the growth was weaker than optimal for sour cabbage micro-aeration. In strict anaerobic during five days were 3 points where gas production was suitable for measurement by apparatus (0.4 L per day). The modelling showed discontinuous hydrogen production as it appeared. In OFR 0.63 mL/h case occurred to change the constant parameters to variables: stress coefficients of bacteria κA, ω, ϙ, stress coefficient of H, S substrate sRate, and hRate, conversion substrate αS with w. The change of hydrogen production parameters z and ξA were more significant in micro-aerobic conditions than in anaerobic. Thus micro-aeration stress bacteria (stress parameters changing) enhanced conversion of a substrate. Therefore, the hydrogen production coefficient z was increasing more than in micro-aerobic conditions. The differences in hydrogen volumes produced in pH 6.0 and pH 7.5 were so huge that it is necessary to divide them into (Figures 3 and 4) (see also Tables 3 and 4 from supplemental materials 20b.pdf). Under these conditions, ensuing parameters were variable in case of pH 6.0 and strictly anaerobic conditions coefficient of change of hydrogen production z, conversion substrate parameters hRate, αS, stress coefficients of bacteria κA, ω, τ ϙ, and ξA. There was a change from constant values to variables from pH 7.5 to 6.0 κA, ω, τ ϙ and hRate. In the case of a pH of 6.0 and micro-aeration, a variable also became sRate (compare Figures 3 and 4 with 5 and 6). These indicated that the conversion of a substrate by bacteria was correlated strongly coefficient of change of hydrogen production z with oxygen presence. The change of pH caused a decrease in the with an increase in stress coefficient ξA changes. The low change of z but changing substrate conversion and stress of bacteria parameters increased hydrogen production efficiency almost 50 times. That shown that manipulating these parameters was relevant variables to optimize the process. The micro-aeration also activated the initial substrate conversion parameter that caused doubling hydrogen production in analogy to strictly anaerobic conditions in low pH. At pH 6.0 The hRate, parameter with time in micro-aeration cases decreased while in anaerobic conditions increased. ξA parameter behaved reversely to hRate. The bacteria stress ω changes were smoother in micro-aeration at pH 6.0 than strictly anaerobic conditions o micro-aeration with neutral pH, (see Figures 5 and 8). Other variables changed faster in the case of micro-aeration. The ϙ value increased in micro-aeration conditions while in anaerobic decreased. Therefore, sRate parameter manipulation allowed changes in the bacteria stress parameters decidedly. Thus, sRate was a more relevant parameter for improving hydrogen production by DF. The supplementary material presented detailed data. Hydrogen production for the semi-batch cabbage dark fermentation was almost two times less than for semi-batch glucose fermentation fed every two days. The stress parameters at the fermentation of glucose were more changing than in the case of sour cabbage. That shows that the simpler the substrate, the more dependents appeared. In the case of complex material, the values of these parameters increased the stability of hydrogen production. Stabilizing of hRate parameter and changing substrate conversion parameter w was caused by boiling of inoculum and low pH (see Tables 7 to 8 (Supplementary materials)). Other parameters changed similarly in micro-aeration, but differences in change of hydrogen production extended. Consequently, there can be observed tripling of hydrogen production, compared to raw inoculum with micro-aeration and low pH similar to [38, 39]. In the case of glucose, the simplification of the substrate caused the stabilization of the αS parameter. The hydrogen production uptake coefficient zz changed in the juxtaposition of glucose DF to the sour cabbage DF. Increasing hydrogen production was higher if feeding was every two days, not three days. Sour cabbage with raw inoculum activated h_Rate_ parameters unlike glucose and sour-cabbage with boiled inoculum. Doubling concentration caused an increase of bacteria growth parameters ϙ and κA and lowering other variables changes in correlation to two days feeding and 5 g VSS/L. Boiling caused those hydrogen bacteria were in endospore form – bacteria were adynamic also. Glucose was a smoothly digested substrate. If microorganisms survived stress and famine, they needed slow recovery with a little portioning of a substrate for overfeeding shock prevention. Thus, bacteria with a less initial concentration of glucose finally produced a higher volume of hydrogen (see, Figure 7). Glucose obtained more hydrogen in similar fermentation conditions than sour cabbage. Simpler glucose was easier digested than sour cabbage. Error analysis showed that the model assessed hydrogen production worse in semi-batch than in the batch process. The error was higher for sour cabbage than for glucose. The complex substrate is more troublesome to model and optimize than glucose. The error was less than 2%, thus acceptable for use [40]. The assessment that hydrogen production was not a continuous process but periodic for one bacteria. One unit of bacteria periodically consumed substrate, reproduced (cell division), and produced hydrogen. Hence for one bacteria unit, that was not a continuous process and could not be described by differential equations. Therefore, analyses were performed at the level of groups of bacterial cells rather than single cells. A continuous DF was observed at the adequate range of bacterial population level and environmental parameters. The hydrogen production had trigonometric characteristics – arcus tangential one. Such analysis can easily lead to misinterpretation and remove data that hydrogen production only starts. Higher initial glucose 10 g VSS/L gave more hydrogen in the period from 4^th^ to 11^th^ days than in 5 VSS/L cases that agree with Kongyan et al. [41, 42]. Besides this period the lower initial substrate concentration resulted in higher cumulative hydrogen production like at Pan et al. works [17, 43]. The Sekoai model [44] for potato waste was using Statistica but did not show the influence of stress. The Akhbari model [19] was similar but did not reveal the origin of formulas like this analysis. The scale-up of DF needs to possess information about points at which hydrogen production started occurring. Until the formulation of a precise enough optimizing procedure, every substrate for a chosen condition needed checking separately. The model proposed in the article can be a step in finding such an optimization approach.

## 5 Conclusions

The hydrogen production was continuous only for the finite large population of bacteria. A single bacteria unit in DF was producing hydrogen periodically. Generally, when some populations were producing hydrogen, and the others were converting substrate, and reversely sometimes all. Therefore, hydrogen production had gaps. After the conversion of a substrate, a metabolism by-product as hydrogen was emitted. Basing on the proposed model of hydrogen was an intermittent phenomenon continuous only for some range. The model was a check for low concentration for sour cabbage and glucose. For glucose DF, the hydrogen production during one month was maximal, 2.45 L, while for sour cabbage DF, 0.9 L.

The assessment that hydrogen production was not a continuous process but periodic for one bacteria. One unit of bacteria periodically consumed substrate, reproduced (cell division), and produced hydrogen. Hence for one bacteria unit, that was not a continuous process and could not be described by differential equations. Therefore, the differential analysis was performed at the level of groups of bacterial cells. A continuous process was observed at an adequate range of bacteria population size and environmental parameters. The hydrogen inhibition or growth depends on the stress of the inoculum. The pH 5.0 seemed to be the most suitable for dark fermentation. The phenomena need further investigation.

## Declarations

### Author contribution statement

Gaweł Sołowski: Conceived and designed the experiments; Performed the experiments; Analyzed and interpreted the data; Contributed reagents, materials, analysis tools or data; Wrote the paper.

Krzysztof Pastuszak: Analyzed and interpreted the data; Contributed reagents, materials, analysis tools or data.

### Funding statement

This work was supported by the 10.13039/501100011755National Center for Research and Development in Poland (project no. BIOSTRATEG 3/344128/12/NCBR/2017), by Opracowanie technologii racjonalnego zagospodarowania strużyn z przetwórstwa skór MIZDRA 2.0 (no POIR.04.01.04-00-0071/20-0), by Wasteman 'Integrated Sustainable Waste Management Systems decreasing pollution discharges in the South Baltic area', and by the National Centre for Research and the Institute of Fluid-Flow Machinery, Polish Academy of Science in Gdansk (grant number FBW-44 – Solowski).

### Data availability statement

Data included in article/supp. material/referenced in article.

### Declaration of interests statement

The authors declare no conflict of interest.

### Additional information

No additional information is available for this paper.

## Appendix A Supplementary data

supplemental materials21b_V2 supplemental materials21b_V2
