# Supplementary material for: Modelling of dark fermentation of glucose and sour cabbage
Source: Heliyon. 2021 Jul 31;7(8):e07690. doi: 10.1016/j.heliyon.2021.e07690 (PMC8350504; doi:10.1016/j.heliyon.2021.e07690)
Supplement: supplemental materials21b_V2 [file mmc1.pdf]

Table 3. Sour cabbage 5 g VSS/L with OFR 0.63 mL/h, raw inoculum, and pH 7.54, batch.

Time [h]	H_2 Total Experiment [L]	H_2 Total Model [%]	Error [%]	z	zz	w	ww	d	h_{Rate}	s_{Rate}	λ	αS	ϱ	ξA	κA	ω	τ
17	0.00327	0.003255	0,37	0.0557	0.0072	-1.9	0.5	1.325	0.51	0.58	0.34	0.85	4.3	0.482	2.62	6.61	2.86
49	0.00421	0.004258	1,212	0.0487	0.072	-1.94	0.5	1.325	0.3	0.465	0.34	0.9	4.9	1.13	3.61	12.26	3.26
93	0.00622	0.006219	-1.7E-06	0.006458	0.072	-1.94	0.5	1.325	0.3	0.515	0.34	0.98	5.1	1.33	3.61	12.11	3.26
115	0.00664	0.00664	-6E-07	0.004121	0.072	-1.94	0.5	1.325	0.3	0.519	0.34	0.98	5.3	1.39	3.61	12.11	3.26

Table 4. Sour cabbage 5 g VSS/L anaerobic with raw inoculum pH 7.54, batch.

Time [h]	H_2 Total Experiment [L]	H_2 Total Model [L]	Error	z	zz	w	ww	d	h_{Rate}	s_{Rate}	λ	αS	ϱ	ξA	κA	ω	τ
17	0.00173	0.00175	-1,273	0.010559	0.072	-1.91	-0.5	1.325	0.31	0.58	0.34	0.81	3.91	0.212	2.62	6.61	2.86
69	0.00207	0.0021	-6.8E-07	0.03249	0.072	-1.91	-0.5	1.325	0.31	0.58	0.34	0.81	3.91	1.13	2.62	6.61	2.86
115	0.00216	0.00214	-2.1E-06	0.000765	0.072	-1.91	-0.5	1.325	0.31	0.58	0.34	0.81	3.91	1.73	2.62	6.61	2.86

Table 5 Sour Cabbage VSS 5 g/L with raw manure batch OFR 0.58 mL/h pH 6.0, batch.

Time [h]	H_2 Total Experiment [L]	H_2 Total Model [L]	Error [%]	z	zz	w	ww	d	h_{Rate}	s_{Rate}	λ	αS	ϙ	ξA	κA	ω	τ
3	0.0288	0.0287	1E-04	0.7545	0.052	-2	0.52	1.24	0.51	0.5	0.34	0.94	1.033	0.06952	1.62	1.61	0.9
4.8	0.041722	0.0407	0.1022	0.5645	0.052	-2	0.52	1.24	0.71	0.5	0.34	0.74	1.133	0.07152	1.62	1.61	0.83
5.1	0.124942	0.1236	0.1342	0.7645	0.052	-2	0.52	1.24	0.51	0.5	0.34	0.94	1.033	0.06952	1.62	1.61	0.9
22	0.209922	0.2098	0.002	0.2448	0.052	-2	0.52	1.24	1.2998	0.5	0.34	0.74	1.133	0.07152	1.62	1.61	0.83
97	0.243522	0.2435	0.002	0.1618	0.052	-2	0.52	1.24	0.81	0.5	0.34	0.94	1.233	0.10652	2.62	2.61	2.36
265	0.271942	0.2719	0.004	0.15295	0.052	-2	0.52	1.24	0.81	0.5	0.34	0.94	1.233	0.10652	2.62	2.61	2.36
289	0.296422	0.2964	0.002	0.1575	0.052	-2	0.52	1.24	0.84	0.52	0.34	0.94	1.233	0.10952	2.62	2.61	2.36
312	0.299279	0.2989	-0.32	0.1575	0.052	-2	0.52	1.24	0.84	0.52	0.34	0.94	1.233	0.10952	2.62	2.61	2.36
358	0.302059	0.3018	0.354	0.1575	0.052	-2	0.52	1.24	0.84	0.52	0.34	0.94	1.233	0.10952	2.62	2.61	2.36
526	0.302628	0.3028	0.003	0.149	0.052	-2	0.52	1.24	0.84	0.52	0.34	0.94	1.233	0.10952	2.62	2.61	2.36

Table 6. Sour Cabbage VSS 5 g VSS/L with raw inoculum anaerobic pH 6.0 batch.

Time [h]	H_2 Total Experiment [L]	H_2 Total Model [L]	Error [%]	z	zz	w	ww	d	h_{Rate}	s_{Rate}	λ	αS	ϙ	ξA	κA	ω	τ
3	0.000179	0.000181	-1,1%	0.1545	0.052	-2	-0.25	1.24	0.91	0.5	0.34	0.94	2.0403	0.13152	1.62	1.61	0.9
24.5	0.059175	0.05884	0,56%	0.1545	0.052	-2	-0.25	1.24	0.91	0.5	0.34	0.94	2.0403	0.13152	1.62	1.61	0.9
222.5	0.094437	0.0937	0,78%	0.061111	0.052	-2	-0.25	1.24	0.791	0.5	0.34	0.82	1.433	0.09552	2.62	2.61	2.36
295.5	0.100285	0.0985	1,8%	0.061321	0.052	-2	-0.25	1.24	0.791	0.5	0.34	0.82	1.433	0.09752	2.62	2.61	2.36
317.5	0.107278	0.1072	0.0078	0.064611	0.052	-2	-0.25	1.24	0.791	0.5	0.34	0.82	1.423	0.09952	2.62	2.61	2.36

380	0.107887	0.1078	0.0085	0.062821	0.052	-2	-0.25	1.24	0.791	0.5	0.34	0.82	1.423	0.09952	2.62	2.61	2.36

Table 7. Sour Cabbage 5 g VSS/L pH 5.0 semi batch OFR 0.8 mL/h feeding every 6 days with 2 g VSS/L.

Time [h]	H_2 Total Experiment [L]	H_2 Total Model [L]	Error [%]	z	zz	w	ww	d	h_{Rate}	s_{Rate}	λ	αS	ϙ	ξA	κA	ω	τ
20	0.0211	0.021	0.47	0.10559	0.072	-1,91	-0.5	1.325	0.31	0.58	0.34	0.81	3.21	0.212	2.62	6.61	
43	0.052791	0.0528	0.57	0.10559	0.072	-1.91	-0.5	1.325	0.31	0.58	0.34	0.81	3.21	0.212	2.62	6.61	2.86
65	0.103975	0.104	0.94	0.12349	0.072	-1.91	-0.5	1.325	0.31	0.62	0.34	0.9	3.91	1.13	3.61	12.21	3.26
79	0.228	0.22914	0.5	0.12349	0.072	-1.91	-0.5	1.325	0.31	0.62	0.34	0.9	3.91	1.13	3.61	12.21	3.26
84	0.256	0.25566	-0.133	0.12349	0.072	-1.91	-0.5	1.325	0.31	0.62	0.34	0.9	3.91	1.13	3.61	12.21	3.26
88	0.397	0.39648	-0.131	0.12349	0.072	-1.91	-0.5	1.325	0.31	0.62	0.34	0.9	3.91	1.13	3.61	12.21	3.26
145	0.4047	0.4042	-0.124	1.3641	0.072	-1.91	-0.5	1.325	0.31	0.63	0.34	0.91	6.35	1.5	3.61	12.31	4.31
217	0.457	0.45503	-0.43	0.48	0.072	-1.91	-0.5	1.325	0.31	0.64	0.34	0.91	7.9	1.6	3.81	12.31	4.66

250	0.605	0.6	-0.826	0.48	0.072	-1.91	-0.5	1.325	0.31	0.64	0.34	0.91	7.9	1.6	3.81	12.31	4.66
322	0.618	0.6164	-0.259	0.485	0.072	-1.91	-0.5	1.325	0.31	0.65	0.34	0.91	12.4	1.62	3.96	12.46	4.76
339	0.624	0.621	-0.3	0.5	0.072	-1.91	-0.5	1.325	0.31	0.65	0.34	0.91	13.9	1.62	3.96	12.46	4.76
342	0.628	0.6272	-0.08	0.5	0.072	-1.91	-0.5	1.325	0.31	0.65	0.34	0.91	13.9	1.62	3.96	12.46	4.76
387	0.645	0.6393	-0.57	0.505	0.072	-1.91	-0.5	1.325	0.31	0.65	0.34	0.92	17.4	1.63	4	12.45	4.77
412	0.695	0.6928	-0.22	0.505	0.072	-1.91	-0.5	1.325	0.31	0.65	0.34	0.92	17.4	1.63	4	12.45	4.77
484	0.751	0.7495	-0.15	0.508	0.072	-1.91	-0.5	1.325	0.31	0.65	0.34	0.91	20.86	1.65	4.1	12.47	4.77
500	0.87	0.863391	-0.66	0.485	0.072	-1.91	-0.5	1.325	0.31	0.65	0.34	0.91	12.4	1.62	3.96	12.46	4.76
543	0.905	0.9001	-0.49	0.485	0.072	-1.91	-0.5	1.325	0.31	0.65	0.34	0.91	12.4	1.62	3.96	12.46	4.76

Table 8. Glucose 5 g VSS/L feeding 2 g VSS/L every 2 days

Time [hours]	H_2 Total Experiment [L]	H_2 Total Model [L]	Error [%]	z	zz	w	ww	d	h_{Rate}	s_{Rate}	λ	αS	ϙ	ξA	κA	ω	τ
20	0.000026	0.000027	- 0.0215	0.51	0.41	-1.9	-0.55	1.625	0.43	0.67	0.03	0.91	616.21	0.89	3.83	5.81	0.93
45	0.000051	0.000051	- 0.86	1.05	0.79	-1.9	-0.55	1.625	0.51	0.56	0.03	0.91	187.21	1.46	42.67	12.81	0.7
72	0.06821	0.0685	0.42	1.65	0.71	-1.92	-0.55	1.625	0.51	0.72	0.03	0.91	32.21	1.96	21.67	12.51	0.7
89	0.07866	0.07718	- 1.7	1.77	0.96	-1.92	-0.55	1.625	0.51	0.72	0.03	0.91	41.21	1.36	21.67	12.51	0.7
93	0.08127	0.08186	- 0.73	1.77	0.96	-1.92	-0.55	1.625	0.51	0.72	0.03	0.91	41.21	1.36	21.67	12.51	0.7
159	0.11498	0.114383	- 0.52	0.0381	0.013	-1.92	-0.55	1.625	0.53	0.65	0.03	0.91	94.31	5.385	17.47	13.21	0.67
164	0.12438	0.12459	- 0.16	0.0381	0.013	-1.92	-0.55	1.625	0.53	0.65	0.03	0.91	94.31	5.385	17.47	13.21	0.67
182	0.1506	0.1512	- 0.36	0.751	0.38	-1.92	-0.55	1.625	0.51	0.62	0.03	0.91	113.12	1.71	19.67	12.51	0.725
208.23	0.17327302	0.172873001	- 0.23	0.3087	0.27	-1.92	-0.55	1.625	0.51	0.615	0.03	0.91	122.22	1.71	18.27	12.21	0.74
229.3	0.25776806	0.25604	0.67	0.25	0.15	-1.92	-0.55	1.625	0.54	0.615	0.03	0.91	89.22	1.79	18.27	12.21	0.74
234	0.28041249	0.2773	1.1	0.25	0.15	-1.92	-0.55	1.625	0.54	0.615	0.03	0.91	89.22	1.79	18.27	12.21	0.74
238	0.56029	0.56729	1.24	0.52	0.35	-1.92	-0.55	1.625	0.51	0.614	0.03	0.91	87.22	1.79	18.27	12.27	0.74
256.5	0.672027749	0.676	- 0.5911	0.52	0.35	-1.92	-0.55	1.625	0.51	0.614	0.03	0.91	87.22	1.79	18.27	12.27	0.74
323.5	1.683471658	1.6839	- 0.0428	0.56	0.25	-1.92	-0.55	1.625	0.51	0.614	0.03	0.91	39.22	1.59	18.1	12.77	0.74
329.5	1.695333366	1.701	- 0.5667	0.56	0.25	-1.92	-0.55	1.625	0.51	0.614	0.03	0.91	39.22	1.59	18.1	12.77	0.74
347	1.719489306	1.71948	0.0009	0.437	0.25	-1.92	-0.55	1.625	0.51	0.674	0.03	0.91	38.62	1.792	18.1	12.27	0.74
359	1.743515096	1.7435	0.0015	0.437	0.25	-1.92	-0.55	1.625	0.51	0.674	0.03	0.91	38.62	1.792	18.1	12.27	0.74
373	1.853307455	1.861	- 0.7693	0.5638	0.26	-1.92	-0.55	1.625	0.53	0.614	0.03	0.91	41.6	1.745	17.21	12.47	0.79
382	1.885826076	1.8866	- 0.0774	0.5638	0.26	-1.92	-0.55	1.625	0.53	0.614	0.03	0.91	41.6	1.745	17.21	12.47	0.79
407	2.070555282	2.069	0.1555	0.762	0.46	-1.92	-0.55	1.625	0.53	0.624	0.03	0.91	46.6	1.785	17.21	12.47	0.89
423	2.349563441	2.351	- 0.1437	0.746	0.46	-1.92	-0.55	1.625	0.53	0.624	0.03	0.91	39.6	1.785	17.21	12.47	0.89
491	2.450301689	2.445	0.53	0.762	0.46	-1.92	-0.55	1.625	0.53	0.624	0.03	0.91	46.6	1.785	17.21	12.47	0.89
